# Anti-Mullerian hormone levels in female cancer patients of reproductive age in Indonesia: A cross-sectional study

**DOI:** 10.12688/f1000research.15728.3

**Published:** 2020-02-20

**Authors:** Achmad Kemal Harzif, Budi Wiweko, Putri Addina, Kartika Iswaranti, Melisa Silvia, Ana Mariana, Kresna Mutia, Kanadi Sumapraja, R Muharam, Gita Pratama

**Affiliations:** 1 Division of Reproductive Endocrionolgy and Infertility, Department of Obstetrics and Gynecology Faculty of Medicine Universitas Indonesia, Dr. Cipto Mangunkusumo Hospital, Jakarta, DKI Jakarta Province, Indonesia; 2 Department of Obstetrics and Gynecology Faculty of Medicine Universitas Indonesia, Dr. Cipto Mangunkusumo Hospital, Jakarta, DKI Jakarta Province, Indonesia; 3 Indonesian Reproductive Medicine Research and Training Center (INA-REPROMED) of Faculty of Medicine Universitas Indonesia,, Dr. Cipto Mangunkusumo Hospital, Jakarta, DKI Jakarta Province, Indonesia

**Keywords:** Anti Mullerian Hormone (AMH), Ovarian Reserve, Cancer in Reproductive, Biological Age

## Abstract

**Background:** Efforts in reproductive preservation for cancer patients have become one of the important aspects of cancer management. In fact, decline in reproductive function is known to occur after exposure to anti-cancer treatments. Measuring anti-Müllerian hormone (AMH) levels is known to be the best parameter in predicting ovarian reserves, which indicates reproductive function. In total, 68% of cancer survivors of reproductive age who underwent anti-cancer treatments suffer from infertility. Meanwhile, ovarian reserves also decrease with increasing age. There is ongoing debate on whether the ovarian reserves of cancer patients could be reduced long before exposure to anti-cancer therapy. Therefore, it is important to know whether ovarian reserves in cancer patients decrease before or after anti-cancer therapy. This can help predict the reproductive function in such cases and the effectiveness of ovarian preservation efforts.

**Methods:** A cross-sectional study was conducted, comparing the AMH levels of 44 female cancer patients of reproductive age before cancer therapy, to 44 non-cancer patients of reproductive age (age matched) *.* The AMH was determined from blood.The biological ages from both groups were adjusted using the Indonesian Kalkulator of Oocytes.

**Results: **The median age in both groups was 28 years old. The AMH levels in the blood of the cancer group were found to be significantly lower in contrast to those in the non-cancer group (1.11 [0.08-4.65] ng/ml vs. 3.99 [1.19- 8.7]; p- value <0.001). Therefore, the biological age in the cancer group was 10 years older than that of the non-cancer group, indicating that ovarian aging occurs earlier in cancer patients.

**Conclusions:** AMH levels of cancer patients of reproductive age were already reduced before cancer therapy, given an older biological age, in contrast to that of the non-cancer patients. Proper counseling and implementation of fertility-preserving methods is highly recommended in this group of patients.

## Introduction

Reproductive preservation efforts for cancer patients have become an important aspect of cancer management. Of all female cancer cases worldwide, 10% of cases occur during reproductive age ^1^. Most cancer patients do not have an opportunity to preserve reproductive function prior to cancer treatment, and decline in reproductive function is known to occur after exposure to cancer treatments, whether it be chemotherapy or radiotherapy. In total, 68% of cancer survivors of reproductive age who have undergone anti-cancer treatments experience infertility, amenorrhea, reduction of ovarian reserves, and premature ovarian failure. Ovarian reserves also decrease with increasing age ^2, 3^, therefore, the American Society of Clinical Oncology (ASCO), and the American Society for Reproductive Medicine (ASRM) have recommended that consultation on fertility preservation methods prior to cancer therapy should be provided ^4^. However, there is an ongoing debate on whether the ovarian reserves of cancer patients could be reduced long before exposure to anti-cancer therapy.

The measurement of the anti-Müllerian hormone (AMH) levels is known to be the best parameter for predicting ovarian reserves, which represent reproductive function ^5^. Low AMH levels reflect a low ovarian reserve ^6, 7^.

AMH is a glycoprotein compound, a member of TGFβ (transforming growth factor) superfamily signaling to specific type II AMH receptor ^8^. AMH was expressed during primary follicles development, and diminishes during maturation of follicle gradually ^9^. In fetal, AMH was secreted by Sertoli cells induce mullerian duct regression leading to female reproductive tract development. Study in AMHKO mice in post natal ovary revealed that AMH was produced by the small growing follicles. The highest level of AMH was detected in preantral and small antral follicles. This finding suggested that AMH correlated with antral follicle count (AFC) ^8^.

Increasing age will decline women's reproductive capacity leading to a decrease in ovarian reserve ^10^. Ovarian reserve decline means that both the size and quality of follicles pool are decreasing. Menopause is constituted by the decline of primordial follicles following by the decrease of AMH levels. Thereby AMH was observed to measure the ovarian reserve, suggested that AMH levels may provide a good test to determine the ovarian reserve in cancer survivors. The Ovarian damage following gonado-toxicity after cancer therapy resulting in loss of primordial follicles ^8^.

Several studies have shown that in breast cancer and lymphoma patients, ovarian reserves are already decreased prior to cancer therapy. However, other studies have shown that a decreased ovarian reserve is only associated with increasing age. It is, therefore, important to know whether the ovarian reserves in cancer patients decrease before or after therapy. This will help predict the reproductive function in such cases and the effectiveness of ovarian preservation efforts.

## Methods

### Study design and patient population

This is a cross-sectional study that compares the AMH levels of reproductive age cancer patients before cancer treatment with non-cancer patients. We enrolled 88 subjects; 44 cancer patients of reproductive age before cancer treatment and 44 non-cancer patients (similar age). For cancer group data, patients were recruited from three places; Obstetrics and Gynecology Polyclinics, Hematology Oncology Polyclinics of Cipto Mangunkusumo Hospital, Obstetrics and Gynecology Polyclinics and Inpatient Unit of Dharmais Cancer Hospital, from May 2015 to December 2017. For non-cancer group data, AMH levels were obtained as secondary data from our previous study ^11^, from which we selected patients with a similar age as patients in the cancer group. Blood samples were collected to measure AMH levels. Inclusion criteria for this study were (a) Cancer and non-cancer patients aged 17–40 years, (b) Cancer patients who have never had a history of cancer therapy: chemotherapy/radiation, de-bulking tumors (specifically gynecology), (c) Non-cancer patients who never had a history of chemotherapy/radiation. No prior pregnancy or infertility treatment for the non-cancer group; and exclusion criteria were (a) respondents who not willing to be a participant in this study, (b) incomplete of filling informed consent and (c) former cancer patient. The sample size was derived from the formula $n=\frac{z^{2}pq}{d^{2}}$ where n is the minimum sample size, Z is the standard normal deviation, as in majority of studies p values are considered significant below 0.05 hence 1.96 is used in formula, p is expected proportion in population based on previous studies (0.36), *q* is 1- *p* (0.64), d is the absolute error or precision (0.1 or 10%). Therefore, the total sample size for this study was calculated to be 88 sample subjects and the comparison of cancer groups before cancer therapy and non cancer group was 1:1 meaning the total sample size for each group was 44 subjects. Unpaired numerical analysis formulas were used to compare AMH levels between cancer patients in reproductive age before cancer therapy with non-cancer patients. The standard deviation for each group are calculated separately, and result shows S1 was standard deviation of the cancer group by: 1,088 ng/dl (mean 1.4 ng/dl) and S2 was standard deviation of non-cancer group by : 2.20 ng/dl (mean 4.37 ng/dl). For calculate the minimum number of samples that can be used in this study, and also know the effect size and power of the study so all collected data were inputted to G Power software version 3.0.10. As a result, minimum sample subject count for each group of at least 7 subjects, with actual P 0.83.

### Data collection

Data collection was performed by consecutive sampling. In cancer group participants, they were given an explanation of the purpose and benefits of the study, and as asked to provide written consent to participate in the study. The written approval sheet was signed by the patient and the researcher. Researchers registered cases and filled out research status forms (extended data) Blood serum (3 ml) from the cubital vein was collected from the cancer group, and stored in vacutainer without anticoagulants. This serum then sent to FKUI Integrated Laboratory for AMH measurement using ELISA techniques by AMH GEN II ELISA Beckman Coulter REF A79765 (with VMax machines). ELISA was performed to the manufacturer’s protocol. After obtaining the results of AMH levels, data analysis was performed. Full method for AMH hormone examination for cancer groups were taken 3 ml of venous blood from the cubital vein of the media and stored in a vacutainer without anticoagulants. After liquefaction and at room temperature, the AMH examination was carried out manually with a micro ELISA technique read with a VMAX type kinetic microplate reader with Softmax Pro Software version 5.4.1.

### Statistical analysis

Statistical analyses were performed using IBM SPSS version 20. Data on ovarian reserves expressed in AMH levels from the cancer patients and the non-cancer patients are presented as medians (minimum-maximum) because the data were not normally distributed. Mann-Whitney tests were performed to compare the differences in ovarian reserves (AMH levels) between the cancer group and the non-cancer group.

### Ethical approval

The Ethics Committee of the Faculty of Medicine from Universitas Indonesia approved this study on May 5, 2015 *(reference number: 286/UN2.F1/ATIK/2015).* All prospective subjects received an explanation from the main researcher and additional researchers regarding the procedures for conducting research. The decision to follow or refuse to follow the research was taken by informed consent. All data will be kept confidential and the subject had the right to know all the results of the examination carried out.

## Results

**Figure 1.**
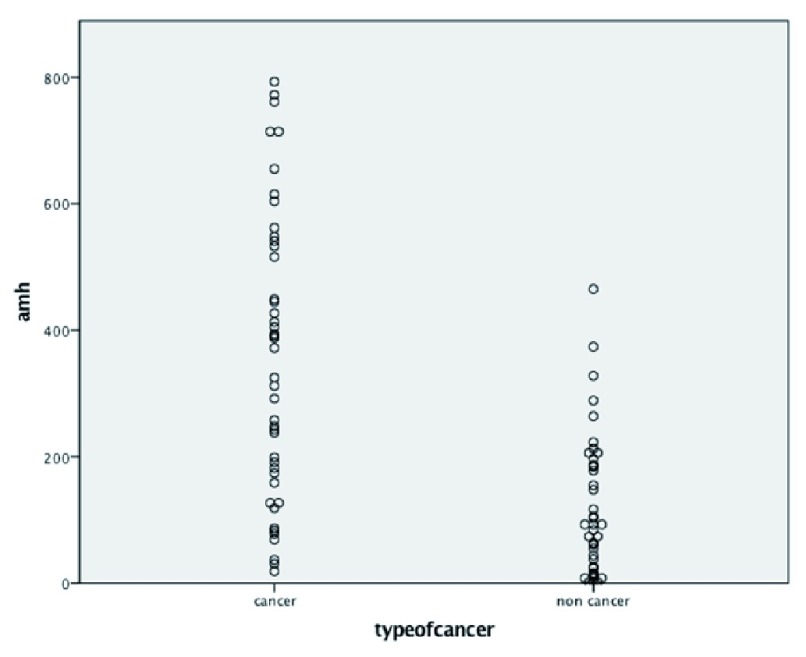
The dot plot of AMH levels distribution between cancer and non-cancer respondents.

The 88 subjects participated in this study, with similar characteristics between the two arms. The mean age for cancer and non-cancer groups was the same, 28 years. For the cancer patient menarche age was 11 years. Body mass index for cancer patients was 20.7 ± 3.89, and non-cancer patients 19.5 ± 2.561. Both groups have the same parity average of 0. The mean AMH level for cancer patients was 1.11 ng/ml (0.08–4.65 ng/ml), and for non-cancer patients 3.99 ng/ml (1.19–8.7 ng/ml). The biological age for cancer patients based on an AMH level of 1.1 ng/ml was 38 years old, and for non-cancer patients 3.99 ng/ml corresponded to 28 years old. Characteristics of research subjects can be seen in Table 1. The dot plot of AMH in all subjects can be seen in Figure 1.

**Table 1.** Baseline characteristics.

Characteristic	Cancer group N: 44	Non-cancer group N: 44	*p-value*
Age, years (min-max)	28 (18–37)	28.5(22–37)	*0.364*
Menarche, age in years (min-max)	11 (8–13)	11 (8–13)	*0.364*
BMI, kg (SD)	20,7 ± 3.89	19,5 ± 2.561	*0.043*
Parity, n (min-max)	0 (0–2)	0 (0–3)	*0.449*
AMH, ng/dl (min-max)	1.11 (0.08–4.65)	3.99 (1.19–8.7)	**<0.001**
Biological age * (min-max)	38	28	**-**

Notes: *Biological age based on AMH level is in accordance with the Indonesian Calculator of Oocyte. BMI: Body mass index, AMH: Anti-Mullerian hormone

Of all the cancer patients recruited in the study, 14 subjects were gynecologic cancer patients and 30 subjects were non-gynecologic cancer patients, the distribution of cancer cases can be seen in Table 2.

**Table 2.** Cancer distribution.

Cancer type	(n)
**Gynecologic cancer**	**14**
Cervical cancer	7
Ovarian cancer	5
Endometrial cancer	1
Vaginal cancer	1
**Non-gynecologic cancer**	**30**
Breast cancer	9
Non-Hodgkin Lymphoma	7
*Acute myelogenous leukemia*	1
*Chronic myelogenous leukemia*	4
*Acute Lymphocytic Leukemia*	2
Rectal cancer	
Tongue cancer	1
Myelodysplastic syndromes	1
Nasopharyngeal cancer	1
Vesical cancer	1
Adenocystic carcinoma	1

Overall, the AMH levels were abnormally distributed (Kolmogorov-Smirnov test for normality, p-value <0.001) with a median level of AMH 2.06 ng/ml (0.08–8.78 ng/ml). AMH levels in the cancer group were lower than that of the non-cancer group (1.11 [0.08–4.65] ng/ml vs. 3.99 [1.19–8.7]; p-value <0.001). This study also showed that there were no statistically significant differences between the AMH levels of the gynecologic cancer patients 0.965 ng/ml (0.08–2.65 ng/ml) and the non-gynecological cancer patients before treatments 1.49 ng/ml (0.08–4.65 ng/ml); p-value 0.162. We found that there is not a significant value for the level of AMH between our ovarian cancer group and the non-ovarian cancer group (0.947). These data may be biased because of the unequal size. Numerous samples are needed to obtain a valid result.

## Discussion

In line with advances in cancer therapy, the survival rate of cancer patients also increases. Unfortunately, this rate of improvement does not coincide with an increased quality of life, especially in regards to reproductive function. Currently, one of the best parameter to measure ovarian reserves is by measuring serum AMH levels; AMH levels can also help predict biological age, which is of more importance when assessing reproductive function.

There are several types of cancer known to be associated with decreased ovarian reserves prior to cancer therapy, such as Hodgkin and Non-Hodgkin’s lymphoma, and breast cancer. Lawrenz *et al*. demonstrated that the AMH levels of lymphoma patients were lower than that in non-cancer patients, with mean AMH levels of 2.06 ng/ml vs. 3.20 ng/dl (p- value <0.05) ^12^. Su *et al*. conducted a similar study wherein the AMH levels of the breast cancer patients were lower than that of the non-cancer patients, 0.6 ng/ml vs. 1.1 ng/ml (p- value <0.001) ^13^. Van *et al*. also conducted a broader study in 2014 comparing the AMH levels in young cancer patients (<18 years) before treatment with non-cancer patients (age-matched). The results showed that AMH levels in the cancer group were significantly lower than that of the non-cancer group, 1.4 mg/l vs. 3.0 mg/l (p- value <0.001) ^14^. The results of these three studies were similar to that of our study; the AMH levels of the 44 reproductive age cancer patients prior to cancer treatment were lower than that of the non-cancer patients (1.11 [0.08–4.65] ng/ml vs. 3.99 [1.19–8.7] ng/ml; p- value <0.001).

Although some studies have shown that the ovarian reserves of reproductive-age cancer patients before treatment are lower than that of the non-cancer patients, the factors that directly affect AMH levels are still unknown. Several studies have suggested genetic mutations, such as BRCA gene mutations, may affect ovarian reserves in breast cancer patients ^15– 17^; the effects of high cytokines in lymphoma patients indirectly affect the ovarian reserves as well ^18, 19^. In non-cancer populations, Jung *et al*. has proven that only the age of menarche affected the concentration of AMH (age of menarche <12 years vs. ≥14 years, 0.90 ng/mL vs. 1.12 ng/mL), while ethnicity, BMI, education level, smoking status, height, and menstrual cycle are not associated with AMH concentrations. There was no significant variations in AMH concentrations between Asian women and white women (age-adjusted model ; p < 0.77, multivariable model ; p < 0.62) ^20^. Bleil *et al*. found there was a statistically significant race/ethnicity-by-linear age interaction among healthy and regularly cycling women, indicating that differences in AMH levels between race/ethnic groups varied as a function of age ^21^. Su *et al*. found that age, BMI, parity, and smoking status are not associated with decreased levels of AMH in patients with breast cancer ^13^. The differences result between previous studies may arise from the varying environment conditions, and heterogeneous character of the populations studied. Serum AMH levels may be influenced by genetic and environmental factors. Our study found that the decreasing level of AMH in cancer group before therapy was related to older of biological age. The result of biological age from both groups were adjusted by Indonesian Kalkulator of Oocytes (IKO) and referred to previous study from Wiweko *et al*. among Indonesian women who went through AMH level test. By using data from this study as reference, we could exclude racial differences of AMH ^22^.

In contrast to other types of non-gynecological cancers, reproductive preservation in gynecological cancers is not widely discussed. This is related to the fact that the genital organs themselves are involved in the cancer and the local treatments of such cancers are destructive to the organs of reproduction ^23^. Therefore, in this study, we compared the AMH levels of gynecologic patients of reproductive age before receiving cancer treatment with the non-gynecological cancer patients, and we found no significant difference between AMH levels (0.965 ng/dl vs. 1.49 ng/dl; p- value 0.162). However, the unequal size of the gynecological and non-gynecological cancers subject groups in our study may have affected the results. With increasing age the capability of a woman to produce appropriate quality oocytes, and quantity of oocytes decreases; this process, called ovarian aging, is defined as a gradual decrease in both the quantity and quality of the oocytes. The decrease in the quality and quantity of these oocytes is related to chronological age and biological age ^24^. Chronological age is determined by the passage of time from birth, while the biological age is determined by physiology ^25^. Biological age affects the reproductive function more than chronological age, besides that ovarian reserve is a good marker for representing the biological age of the ovary. In this study, the biological age of the cancer group was 10 years higher than that of the non-cancer group, indicating that ovarian aging occurs earlier in cancer patients. This would indicate that reproductive preservation methods should be offered long before cancer patients undergo therapy.

There is a hypothesis that reported a decreased number of ovarian follicles and an increased gonadotropin level which caused an inflammatory environment and changing epithelial cell surface and the development of tumors in the ovary ^26^. Other studies have suggested genetic mutations were associated with ovarian aging. Johnson *et al*. observed BRCA2 carriers had significantly lower AMH levels compared to healthy, low-risk women and had increased odds of having a low AMH (OR 3.69, 95% 1.34–10.19, p=0.012). BRCA 2 as tumor suppressor genes which involved in the regulation of follicular pool through impairment of DNA repair pathway and affected ovarian reserves in breast cancer patients ^27^. A study in lymphoma patients showed significantly lower AMH levels than in the control group. There is a strong negative correlation between AMH with SIL-2R, IL-6, and IL-8 cytokines exists ^18^. The impairment of DNA repair mechanisms may affect the granulosa cell function. This condition cause AMH decreased independently in childhood cancer ^14^.

There are many reproductive preservation techniques; however, one of technique for cancer patients is ovarian cryopreservation. This technique consists of two methods: the slow cooling method and vitrification ovary. Based on the research conducted by Wiweko *et al*., ovarian vitrification techniques are superior to the slow cooling method ^28^. Ovarian vitrification does not alter the morphology of the granulosa cells, or the stromal and ovarian collagen components ^22^. It has also been shown to not change the morphology of the pre-antral follicle nor does it increase the risk of cell apoptosis ^22, 28^. Other options that may still be offered to cancer patients of reproductive age are oocyte cryopreservation and embryo cryopreservation. In patients who already have a partner or are married, embryo cryopreservation can be offered, however, this may delay cancer therapy due to the ovarian stimulation process required for this technique. In addition to this, estrogen exposure given at the time of ovarian stimulation may have an adverse effect in estrogen sensitive tumors. Unlike embryo cryopreservation, cryopreservation of the oocytes can be used for single women who can undergo the stimulation cycle, but the effectiveness of this technique is very low. The pregnancy and delivery rates range from 1 to 5% per frozen oocyte ^29^. The current weakness of this study is that the other gynecological abnormalities that may affect AMH levels in both groups were not included in the screening criteria for exclusion, such as genetic factors and the type/stage of cancer or other diseases which may affect the levels of AMH, were also not studied.

## Conclusions

This study showed that the AMH levels in reproductive age patients before receiving therapy were lower in contrast to that in the non-cancer patients (1.11 ng/ml vs. 3.99 ng/ml; p- value <0.001). In addition to this, there was no significant difference in the AMH levels between the gynecologic and non-gynecologic cancer groups before treatment (0.965 ng/dl vs. 1.49 ng/dl; p- value 0.162). There was no significant difference of AMH levels between ovarian cancer and non-ovarian cancer (p-value 0.947).

## Data availability

### Underlying data

All raw data and demographic information obtained from subject during the present study, Dataset: Anti-Mullerian hormone levels in female cancer patients of reproductive age in Indonesia: A cross-sectional study. figshare. Dataset. https://doi.org/10.6084/m9.figshare.11729031.v1 ^30^

### Extended data

Figshare: Anti-Mullerian hormone levels in female cancer patients of reproductive age in Indonesia: A cross-sectional study, Dataset. https://doi.org/10.6084/m9.figshare.11729004.v1

This project contains the following extended data:

- Research Questionnaire in English contains the English version of questionnaire which used in this study.

- Research Questionnaire in Bahasa Indonesia contains the Bahasa version of questionnaire which used in this study.

Data are available under the terms of the Creative Commons Attribution 4.0 International license (CC-BY 4.0).
