# Supplementary material for: Anti-Mullerian hormone levels in female cancer patients of reproductive age in Indonesia: A cross-sectional study
Source: F1000Res. 2020 Feb 20;8:159. Originally published 2019 Feb 6. [Version 3] doi: 10.12688/f1000research.15728.3 (PMC7059784; doi:10.12688/f1000research.15728.3)
Supplement: Peer review history

# Peer review: Reviewer response for version 3

**DOI:** 10.5256/f1000research.24255.r60313

**Published:** 2020-03-05

**Authors:** Xiang-Xi (Mike) Xu

**Affiliations:** 1 Sylvester Cancer Center (SCCC), Ovarian Cancer Program, University of Miami, Miami, FL, USA

I am satisfied with the revision

I confirm that I have read this submission and believe that I have an appropriate level of expertise to confirm that it is of an acceptable scientific standard.

# Peer review: Reviewer response for version 2

**DOI:** 10.5256/f1000research.21664.r54585

**Published:** 2019-10-02

**Authors:** Xiang-Xi (Mike) Xu

**Affiliations:** 1 Sylvester Cancer Center (SCCC), Ovarian Cancer Program, University of Miami, Miami, FL, USA

The study is interesting and the conclusion is provocative: AMH is 1.11 in cancer and 3.99 in non-cancer. However, there are several red flags and suggestions need to be considered:

  

1. It should be mentioned that AMH was determined from blood, in Abstract and Results.
2. If the authors have sufficient background, it should be discussed about the biology and AMH production by ovarian follicles.
3. The number of patients is not very big. It would be more informative to see a dot plot where every patient represents one dot.
4. The Age of the patients (28, 18-37) is something of a red flag. For ovarian epithelial cancer, 95% of the cases are 55 or older.
5. Doubtfully, all cancer types are affected by AMH. The gynec cancer (14 cases) should be also analysed separately.

I confirm that I have read this submission and believe that I have an appropriate level of expertise to confirm that it is of an acceptable scientific standard, however I have significant reservations, as outlined above.

# Response

**Published:** 2020-01-08

**Authors:** Achmad Kemal

**Affiliations:** RSUPN Dr. Cipto Mangunkusumo Hospital, Indonesia

Dear Xiang-Xi,

Thank you for the review. We have uploaded the revision of our manuscript regarding to your suggestion.

**1. It should be mentioned that AMH was determined from blood, in Abstract and Results.**

*Yes, we agree with your suggestion to mentioned that AMH was determined from blood.*

*The change has been made.*

**2. If the authors have sufficient background, it should be discussed about the biology and AMH production by ovarian follicles.**

*We have added some references about AMH production* 

**3. The number of patients is not very big. It would be more informative to see a dot plot where every patient represents one dot.**

*The dot plot of age, biological age, menarche, parity, and AMH in all subjects can be seen in Figure 1.*

**4** ***.*** **The Age of the patients (28, 18-37) is something of a red flag. For ovarian epithelial cancer, 95% of the cases are 55 or older.**

*In this study, we collected samples between 17 and 40 yr to measure the level of AMH that correlated with reproductive age and compared their biological and chronological age. We have found a significant result that female cancer patients are highly potential to have ovarian aging earlier than non-cancer patients .*

**5. Doubtfully, all cancer types are affected by AMH. The gynec cancer (14 cases) should be also analysed separately.**

*We found that there is not a significant value for the level of AMH between our ovarian cancer group and the non-ovarian cancer group (0.947). These data may be biased because of the unequal size. Numerous samples are needed to obtain a valid result.*

We carefully have modified the manuscript according to your instructions. Thank you for your considerations, we looking forward to any helpful feedback.

Best Regards,

Achmad Kemal

# Response

**Published:** 2020-01-27

**Authors:** Achmad Kemal

**Affiliations:** RSUPN Dr. Cipto Mangunkusumo Hospital, Indonesia

Dear Mike Xu,

We hope that you can give us further feedback and evaluation according to your queries

Achmad Kemal Harzif

# Peer review: Reviewer response for version 2

**DOI:** 10.5256/f1000research.21664.r51171

**Published:** 2019-07-22

**Authors:** Nao Suzuki

**Affiliations:** 1 Department of Obstetrics and Gynecology, St. Marianna University School of Medicine, Kawasaki, Japan

I think the author's revisions are sufficient for me to amend the status of 'approved with reservations'.

I confirm that I have read this submission and believe that I have an appropriate level of expertise to confirm that it is of an acceptable scientific standard.

# Peer review: Reviewer response for version 1

**DOI:** 10.5256/f1000research.17166.r48874

**Published:** 2019-06-10

**Authors:** Nao Suzuki

**Affiliations:** 1 Department of Obstetrics and Gynecology, St. Marianna University School of Medicine, Kawasaki, Japan

This is a well-written manuscript about Anti-Mullerian hormone levels in female cancer patients of reproductive age in Indonesia. The aim of the study is to predict the reproductive function of cancer patients. The major limitation of the study is a low number of participants in two groups. I have a few other comments for the authors.

- Page 3 left panel L 21: I think the AMH levels are known to be one of the best parameters for predicting ovarian reserves. I do not think this is the best parameter. I need the author's opinions.
- Page 3 left panel L 43: Please add the information about the past history of pregnancy and birth, and infertility treatment in the non-cancer group.
- Whether there is racial difference? Please add the author's opinions in the discussion.
- Please add more precise opinions regarding the reasons why ovarian aging occurs earlier in cancer patients.

I confirm that I have read this submission and believe that I have an appropriate level of expertise to confirm that it is of an acceptable scientific standard, however I have significant reservations, as outlined above.

# Response

**Published:** 2019-06-19

**Authors:** Achmad Kemal

**Affiliations:** RSUPN Dr. Cipto Mangunkusumo Hospital, Indonesia

We are grateful for the helpful feedback by the reviewer that helped us improve the quality of our manuscript. We carefully responded to all points and have modified the manuscript accordingly. Please see below our detailed response to comments.

**Page 3 left panel L 21: I think the AMH levels are known to be one of the best parameters for predicting ovarian reserves. I do not think this is the best parameter. I need the author's opinions.**

 Answer: Yes, we agree that AMH level is one of the best parameters for predicting ovarian reserves. The correction has been made in the manuscript

**Page 3 left panel L 43: Please add the information about the past history of pregnancy and birth, and infertility treatment in the non-cancer group.**

 Answer:

 We have added the information for the inclusion and exclusion criteria. The highlighted sentences are the additional information. “…Inclusion criteria for this study were (a) Cancer and non-cancer patients aged 17–40 years (b) Cancer patients who have never had a history of cancer therapy: chemotherapy/radiation, de-bulking tumors (specifically gynecology) **(c) Non-cancer patients who never had a history of chemotherapy/radiation. No prior pregnancy or infertility treatment for the non-cancer group**; and exclusion criteria were (a) respondents who not willing to be a participant in this study (b) incomplete of filling informed consent and (c) former cancer patient.

 The informations have been added to the manuscript.

**Whether there is racial difference? Please add the author's opinions in the discussion**

Answer:

 We thank the reviewer for this comment. As explained in Page 5 left panel L 45, Jung et al found that there was no significant variations in AMH concentrations between Asian women and white women (age-adjusted model ; p < 0.77 , multivariable model ; p < 0.62). Although, Bleil et al. found there was a statistically significant race/ethnicity-by-linear age interaction among healthy and regularly cycling women, indicating that differences in AMH levels between race/ethnic groups varied as a function of age. The differences result between previous studies may arise from the varying environment conditions, and heterogeneous character of the populations studied. Serum AMH levels may be influenced by genetic and environmental factors.

 Our study found that the decreasing level of AMH in cancer group before therapy was related to older of biological age. The result of biological age from both groups were adjusted by Indonesian Kalkulator of Oocytes (IKO) and referred to previous study from Wiweko et al among Indonesian women who went through AMH level test. By using data from this study as reference, we could exclude racial differences of AMH.

 Our opinions have been added in the discussion of this manuscript.

**Please add more precise opinions regarding the reasons why ovarian aging occurs earlier in cancer patients.**

 Answer:

 There is a hypothesis that reported a decreased number of ovarian follicles and an increased gonadotropin level which caused an inflammatory environment and changing epithelial cell surface and the development of tumors in the ovary. Other studies have suggested genetic mutations were associated with ovarian aging. Johnson et al observed BRCA2 carriers had significantly lower AMH levels compared to healthy, low-risk women and had increased odds of having a low AMH (OR 3.69, 95% 1.34–10.19, p=0.012). BRCA 2 as tumor suppressor genes which involved in the regulation of follicular pool through impairment of DNA repair pathway and affected ovarian reserves in breast cancer patients. A study in lymphoma patients showed significantly lower AMH levels than in the control group. There is a strong negative correlation between AMH with SIL-2R, IL-6, and IL-8 cytokines exists. Van Dorp et al found that a significantly lower serum AMH levels of girls with childhood cancer compared with healthy age-matched controls (standard deviation scores (SDS) 20.8, p<0.001). Potential cause of the decreased AMH independently of childhood cancer subtype might be impaired granulosa cell function due to an impaired DNA repair mechanism.

 Our opinions have been added to the manuscript. We carefully have modified the manuscript according to the editor instructions. Thank you for your considerations, we looking forward to any helpful feedback.

 Best Regards,

 Achmad Kemal
